# Conversion to Sinus Rhythm in Refractory Atrial Fibrillation Patients after Atrioventricular Node Ablation with Conduction System Pacing

**DOI:** 10.31083/j.rcm2411333

**Published:** 2023-11-24

**Authors:** Maja Ivanovski, Miha Mrak, Anja Zupan Mežnar, David Žižek

**Affiliations:** ^1^Department of Cardiology, University Medical Centre Ljubljana, 1000 Ljubljana, Slovenia; ^2^Faculty of Medicine, University of Ljubljana, 1000 Ljubljana, Slovenia

**Keywords:** atrial fibrillation, conduction system pacing, left bundle branch pacing, His bundle pacing, atrioventricular node ablation, sinus rhythm

## Abstract

**Background:**

“Ablate and pace” strategy is a reasonable treatment option in refractory atrial fibrillation (AF) when sinus rhythm (SR) cannot be achieved with catheter ablation or pharmacological therapy. Atrioventricular node ablation (AVNA) combined with conduction system pacing (CSP), with left bundle branch pacing (LBBP) or His bundle pacing (HBP), is gaining recognition since it offers the most physiological activation of the left ventricle. However, the incidence of conversion to SR after AVNA with CSP is not known. The purpose of the investigation was to determine the incidence of spontaneous conversion to SR and its predicting factors in patients undergoing CSP and AVNA.

**Methods:**

Consecutive refractory symptomatic AF patients undergoing AVNA with CSP at our institution between June 2018 and December 2022 were retrospectively analyzed. Twelve lead electrocardiogram (ECG) recordings were analyzed at each outpatient follow-up visit. Echocardiographic and clinical parameters were assessed at baseline and six months after the implantation.

**Results:**

Sixty-eight patients (male 42.6%, age 71 ± 8 years, left ventricular ejection fraction 40 ± 15%) were included. Thirty-seven patients (54.4%) received HBP and 31 (45.6%) LBBP. During follow-up, spontaneous conversion to SR was registered in 6 patients (8.8%); 3 in the HBP group and 3 in the LBBP group. Baseline characteristics of patients who converted to SR did not differ from non-sinus rhythm (NSR) patients except for left atrial volume index (LAVI), which was significantly smaller in the SR group (45 mL/$m^{2}$ (41–51) vs. 60 mL/$m^{2}$ (52–75); *p* = 0.002). Multiple regression model confirmed an inverse association between LAVI and conversion to SR even after considering other clinically relevant covariates (odds ratio 1.273, *p* = 0.028). At follow-up, LAVI did not change in any group (SR: *p* = 0.345; NSR: *p* = 0.508). Improvement in New York Heart Association (NYHA) class was comparable in both groups.

**Conclusions:**

Spontaneous conversion to SR after AVNA combined with CSP is not uncommon, especially in patients with smaller left atria. Further studies are warranted to clarify which patients should be considered for initial dual-chamber device implantation to provide atrio-ventricular synchrony in case of SR restoration.

## 1. Introduction 

Atrial fibrillation (AF), the most prevalent supraventricular tachyarrhythmia (SVT), results in disorganized atrial activity, reduced cardiac output, and hemodynamic deterioration. The progressive nature of AF has been attributed to alterations in the electrical, contractile, and structural properties of the atria. Some of these changes appear to be reversible upon the improvement of hemodynamics [1, 2, 3].

There are two basic approaches for the treatment of AF: rate and rhythm control. Antiarrhythmic drugs and/or pulmonary vein isolation (PVI) are used to maintain sinus rhythm (SR) as part of rhythm control management. The guidelines for the management of SVT recommend atrioventricular node ablation (AVNA) with permanent ventricular pacing (‘pace and ablate’ strategy) when SR is no longer pursued or attainable (Class I, level of evidence C) [4]. AVNA with subsequent right ventricular (RV) or biventricular (BiV) pacing in these patients results in symptomatic improvement, reduced heart failure (HF) hospitalizations, and improved survival [5, 6]. In addition, analysis of retrospective data raises the possibility that a rate control strategy with BiV pacing may even contribute to spontaneous SR restoration [7, 8, 9]. As BiV pacing still causes non-physiologic cardiac activation in patients with narrow QRS [8, 9, 10, 11], left bundle branch pacing (LBBP) and His bundle pacing (HBP) have recently evolved as conduction system pacing (CSP) options allowing more physiological activation of the myocardium which preserves left ventricular (LV) function [12, 13, 14, 15, 16, 17, 18].

The incidence of SR restoration after the “ablate and pace” strategy with CSP has been unexplored. The purpose of the investigation was to determine the incidence of spontaneous conversion to SR and its predicting factors in patients with refractory AF undergoing AVNA and CSP.

## 2. Materials and Methods

### 2.1 Study Design

This study retrospectively investigated the incidence of spontaneous conversion to SR and its predicting factors in patients who underwent attempted AVNA and CSP for symptomatic AF between June 2018 and December 2022 at University Medical Centre Ljubljana. Consecutive patients with symptomatic AF refractory to rhythm and pharmacological rate control therapy were included. Patients with severe valvular disease were excluded. SR restoration was defined as the spontaneous conversion to SR documented on 12-lead electrocardiogram (ECG) in the device clinic during follow-up after AVNA and CSP.

All patients were asked for written informed consent before data collection. The study design was approved by the Republic of Slovenia National Medical Ethics Committee.

### 2.2 Procedures

Pacing device implantations were always performed first, followed by AVNA, preferably during the same hospitalization. All device implantations were performed by two experienced operators.

#### 2.2.1 His Bundle Pacing

**Fig. 1.**
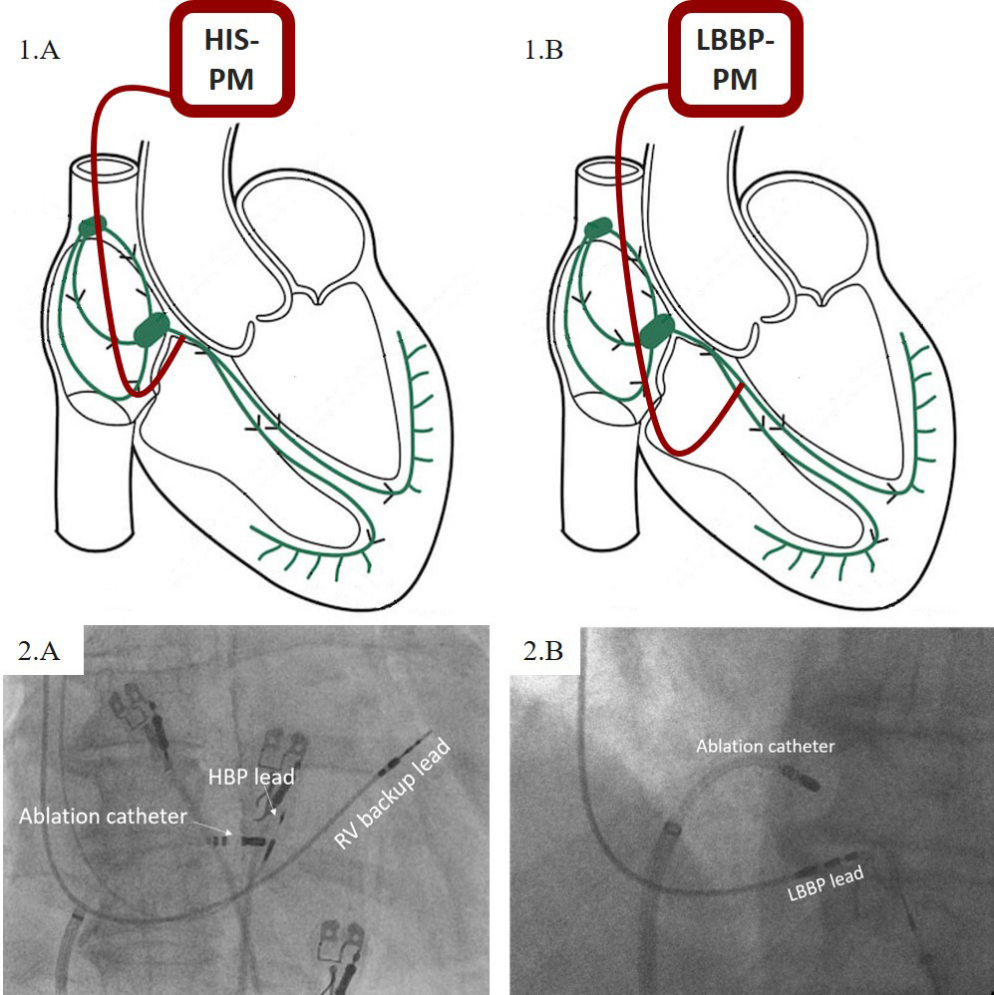
**Schematic representation of pacemaker (PM) lead positions (1) and their relation to the ablation catheter under fluoroscopy (2)**. (A) His bundle pacing (HBP). (B) Left bundle branch pacing (LBBP). For clarity, the backup pacing lead is not illustrated.

The HBP procedures were conducted as previously reported [13, 19, 20]. His bundle (HB) potential mapping was performed using a continuous recording of intracardiac electrograms with the electrophysiological recording system (EP-TRACER 2 Portable CardioTek B.V., Sittard, The Netherlands or LAB system Pro, BARD Boston Scientific, Lowell, MA, USA). The tricuspid valve annulus ring was imaged by contrast angiography to facilitate HB localization. The sheath and the pacing lead were advanced to the HB area, where larger ventricular and smaller atrial signals were detected (ventricular to atrial electrogram ratio at least 3:1). The pacing lead was screwed into position, and threshold measurement was performed at the pulse width of 1 ms. HBP threshold ≤2.5 V at a pulse width of 1.0 ms was considered appropriate. Additional backup pacing or implantable cardioverter-defibrillator (ICD) lead was implanted and connected to the ventricular port of the pacing device or IS-1 port of a DF-1 ICD device, while HBP lead was connected to the atrial port of the dual-chamber device. Schematic illustration and fluoroscopic view of the pacemaker lead position are presented in Fig. 1A.

#### 2.2.2 Left Bundle Branch Pacing 

The procedure was performed as previously described [21]. After the localization of the HB area, either fluoroscopically or determined with the use of intracardiac signals, the LBBP lead was positioned approximately 1–1.5 cm below the distal HB location along the line towards the RV apex. Following the optimal lead positioning with the use of fluoroscopy and paced QRS morphology (“w” pattern in lead V1), the lead was screwed into the interventricular septum with constant monitoring of pacing impedance, current of injury, and QRS morphology. Trans-septal lead advancement was stopped when typical left bundle branch capture morphology was reached. Both lumen-less and stylet-driven leads were used for LBBP. While backup pacing leads were never implanted due to low and stable pacing parameters, the ICD lead, if needed, was connected in the same fashion as in HBP procedures [22]. Schematic illustration and fluoroscopic view of the pacemaker lead position are presented in Fig. 1B.

#### 2.2.3 Atrioventricular Node Ablation

Prior to AVNA, the previously implanted pacemaker was temporarily set to 40 bpm for the duration of the procedure. After achieving femoral vein access, the ablation catheter with a 3.5- or 4-mm tip (${\mathrm{FlexAbility}}^{\text{TM}}$, Abbott, Abbott Park, IL, USA or CelsiusVR ThermocoolVR, BiosenseWebster, Irvine, CA, USA) was advanced through a long sheath. Ablation was initially targeted at the mid-septum under fluoroscopy. The location was optimized according to the intracardiac electrograms. In the case of HBP, the ring of the pacing lead was used as a target zone to provide a safe distance and prevent the rise of the threshold following AVNA. Ablation was performed in a temperature-controlled mode (40 W, up to 60 seconds). Successful AVNA was recognized as an abrupt and persistent drop in heart rate. After AVNA, the base rate was initially set to 80 or 90 bpm (depending on the baseline ventricular rate). We decreased the base rate to 70 bpm after a 1-month of follow-up [15, 16, 23].

### 2.3 Outcomes and Device Follow-Up

Twelve-lead ECGs were assessed at baseline and each outpatient follow-up visit: at 1 month, 6 months, and every 6 months thereafter. Assessment of clinical outcomes (echocardiographic parameters, laboratory parameters, and symptomatic evaluation) was performed at baseline and at 6 months or immediately after SR on the ECG strip was detected.

### 2.4 Statistical Analyses

Categorical variables are reported as frequencies and percentages and were compared using Chi-square and Fisher exact test as appropriate. Continuous variables are expressed as mean ± standard deviation or as median (interquartile range), according to the distribution. Kolmogorov–Smirnov test was used to test the normality of distribution. Intra- and intergroup differences were compared with the use of independent or paired sample Student *t*-test, Wilcoxon rank-sum test, and Wilcoxon signed-rank test as appropriate. The multivariate logistic regression model was used to analyze predictors of spontaneous conversion to sinus rhythm. All hypotheses were two-tailed, and a *p*-value ≤ 0.05 was considered significant. Statistical analysis was performed in IBM SPSS Statistics for Windows, version 25.0 (IBM Corp., Armonk, NY, USA).

## 3. Results

### 3.1 Patient Characteristics

Baseline patient characteristics according to the occurrence of spontaneous conversion to SR are presented in Table 1. Sixty-eight consecutive patients undergoing CSP combined with AVNA were included. The mean age of the patients was 71 ± 8 years, and 29 (42.6%) were male. The baseline QRS width was 128 ms (110–140), and twelve patients (17.6%) had atypical atrial flutter. Patients were highly symptomatic with the median New York Heart Association (NYHA) class 3. The mean left ventricular ejection fraction (LVEF) was reduced to 40 ± 15%, and the atria were enlarged with the median left atrial volume index (LAVI) of 59 mL/$m^{2}$ (51–72). Thirty-seven patients (54.4%) received HBP and 31 (45.6%) LBBP.

**Table 1.** **Baseline characteristics of patients by conversion to sinus rhythm**.

		SR (n = 6)	NSR (n = 62)	*p*-value
Baseline characteristics				
Age [years]		70 (±10)	71 (±8)	0.673
Male sex		2 (33.3%)	27 (43.5%)	1
QRS [ms]		108 (94–119)	109 (95–120)	0.792
	LBBB	0	6 (9.7%)	1
	RBBB	0	2 (3.2%)	1
Atypical atrial flutter		1 (16.7%)	11 (17.7%)	1
LVEF [%]		40 (±15)	40 (±16)	1
LAVI [mL/$m^{2}$]		45 (41–51)	60 (52–75)	**0.002**
Initial NYHA class		3.5 (3–4)	3 (3–3)	0.082
Arterial hypertension		4 (66.6%)	45 (72.6%)	1
Diabetes		1 (16.7%)	17 (27.4%)	1
Coronary artery disease		1 (16.7%)	13 (21%)	1
Medications				
Loop diuretic		3 (50%)	39 (62.9%)	0.668
ACEi/ARB/ARNI		2 (33.3%)	40 (64.5%)	0.193
MRA		0	24 (38.7%)	0.083
Beta-blocker		5 (83.3%)	59 (95.1%)	0.315
Digoxin		1 (16.7%)	16 (25.8%)	1
Amiodarone		2 (33.3%)	15 (24.2%)	0.635

Legend: SR, sinus rhythm group; NSR, non-sinus rhythm group; LBBB, left bundle branch block; RBBB, right bundle branch block; LVEF, left ventricular ejection fraction; LAVI, left atrial volume index; ACEi, angiotensin-converting enzyme inhibitor; ARB, angiotensin II receptor blocker; ARNI, angiotensin receptor neprilysin inhibitor; MRA, mineralocorticoid receptor antagonist; NYHA, New York Heart Association. Bold value denotes statistical significance at the *p* < 0.05 level.

### 3.2 Predicting Factors for Conversion to Sinus Rhythm 

The median follow-up time was 16 months (6–27). Spontaneous conversion to SR during follow-up was registered in 6 patients (8.8%); 3 in the HBP group and 3 in the LBBP group. In patients who converted to SR, baseline LAVI was smaller (45 mL/$m^{2}$ (41–51) vs. 60 mL/$m^{2}$ (52–75); *p* = 0.002) (Table 1). Due to cardiogenic shock, one of the SR patients required temporal circulatory support with veno-arterial (VA) extracorporeal membrane oxygenation (ECMO), which was applied 24 hours before pacemaker implantation and AVNA. SR on ECG was first detected after the median follow-up of 4.5 months (2–24).

To further clarify the predictors of spontaneous conversion to SR after AVNA, we performed a multiple regression analysis (Table 2). Covariates that were considered clinically relevant were age, gender, baseline LVEF, LAVI, and indexed left ventricular end-diastolic volume index (LVEDVi). Even after consideration of these covariates, LAVI remained a significant predictor for conversion to SR (odds ratio 1.273, 95% confidence interval [1.027, 1.578], *p* = 0.028).

**Table 2.** **Multivariate logistic regression model showing predictors of spontaneous conversion to sinus rhythm after atrioventricular node ablation and conduction system pacing (reference category = sinus rhythm)**.

	OR (95% CI)	*p*-value
Sex	0.315 (0.028, 3.585)	0.352
Age	1.022 (0.899, 1.162)	0.738
Initial LVEF [%]	1.117 (0.960, 1.300)	0.151
Initial LVEDVi [mL/$m^{2}$]	1.112 (0.976, 1.267)	0.111
Initial LAVI [mL/$m^{2}$]	1.273 (1.027, 1.578)	**0.028**

Legend: OR, odds ratio; CI, confidence interval; LVEF, left ventricular ejection fraction; LVEDVi, left ventricular end-diastolic volume index; LAVI, left atrial volume index. Bold value denotes statistical significance at the p < 0.05 level.

### 3.3 Procedural Outcomes and Complications

All device implantations and subsequent AVNAs were successful without any acute adverse events. Apart from two patients in whom an existing ICD device was upgraded with a CSP lead, all other procedures were de-novo implantations. The median pacemaker implantation fluoroscopy time was 6 minutes (4.2–8.1). An ICD device for primary prevention was used in 3 (8%) patients who received HBP and 7 (22%) patients who underwent LBBP. Additional atrial lead was implanted in only one patient. Periprocedural increase in HBP threshold after AVNA was documented in 1 patient. However, the HB capture was maintained at higher outputs, and the lead revision was not required.

### 3.4 Clinical and Laboratory Outcomes

At baseline, NYHA class of patients who converted to SR (SR group) did not differ from patients who remained in atrial arrhythmia (non-sinus rhythm (NSR) group) (*p* = 0.082). As reported in Table 3, NYHA class improvement was registered regardless of SR restoration (*p* = 0.026 for SR; *p* < 0.001 for NSR). In the SR group, 2 patients improved for 1 NYHA class, 3 patients improved for 2 NYHA classes, and 1 patient improved for 3 NYHA classes. In the NSR group, 27 patients improved for 1 class, 19 patients improved for 2 classes, and 1 patient improved for 3 NYHA classes. No change in NYHA class was detected in 15 patients. No patient deteriorated in any group. In all patients, digoxin and amiodarone were discontinued, and dosages of beta-blockers were reduced. In the NSR group, diuretics were discontinued in 15 of 39 patients who were receiving them at baseline (28.0%). In the SR group, they were discontinued in 1 of 3 patients (33.3%); however, the difference did not reach statistical significance. Other HF therapy did not change during follow-up.

**Table 3.** **Comparison of clinical and laboratory parameters of patients according to sinus rhythm conversion at baseline and during follow-up**.

		SR (n = 6)	NSR (n = 62)	*p*-value — comparing groups
NYHA class				
Baseline NYHA class		3.5 (3–4)	3 (3–3)	0.082
	Nb. in NYHA class 2	0	14 (22.6%)	
	Nb. in NYHA class 3	3 (50%)	37 (59.7%)	
	Nb. in NYHA class 4	3 (50%)	11 (17.7%)	
Follow-up NYHA class		1.5 (1–2)	2 (1–2)	0.419
	Nb. in NYHA class 1	3 (50%)	16 (25.8%)	
	Nb. in NYHA class 2	2 (33.3%)	39 (62.9%)	
	Nb. in NYHA class 3	1 (16.7%)	7 (11.3%)	
	Nb. in NYHA class 4	0	0	
*p*-value: baseline vs. follow-up		**0.026**	< **0.001**	
Loop diuretics				
Baseline		3 (50%)	39 (62.9%)	0.668
Follow-up		2 (33.3%)	27 (43.5%)	1
*p*-value: baseline vs. follow-up		1	**0.047**	
NT-proBNP [pg/mL]				
Baseline (n = 61)		5122 (2800–12,059)	2894 (1552–7285)	0.339
Follow-up (n = 55)*		1437 (1042–2229)	2034 (976–3001)	0.599
*p*-value: baseline vs. follow-up		0.625	< **0.001**	
eGFR [mL/min/1.73 $m^{2}$]				
Baseline (n = 66)		54 (32–67)	52 (43-63)	0.240
Follow-up (n = 60)*		54 (49–90)	67 (46-75)	0.214
*p*-value: baseline vs. follow-up		0.688	< **0.001**	

Legend: SR, sinus rhythm group; NSR, non-sinus rhythm group; NYHA, New York Heart Association; NT-proBNP, N-terminal pro-b-type natriuretic peptide; eGFR, estimated glomerular filtration rate. * 7 patients in the NSR group and 1 in the SR group did not have follow-up GFR values, 11 patients in NSR and 1 patient in the SR group did not have NT-proBNP values at follow-up. Bold values denote statistical significance at the *p* < 0.05 level.

At baseline, median N-terminal pro-b-type natriuretic peptide (NT-proBNP) was 2969 (1569–3635) pg/mL and did not differ between both groups (*p* = 0.339). At follow-up, NT-proBNP decreased in both groups, although the decrease in the SR group, despite being relatively higher, did not reach statistical significance due to the smaller sample size.

Four patients in the NSR group (3 with HBP and one with LBBP) died during follow-up. While the death in the LBBP group was associated with progressive HF, the other 3 deaths were determined as non-cardiac.

### 3.5 Electrocardiographic and Echocardiographic Outcomes

At baseline, 6 patients had left bundle branch block (LBBB), and 2 patients had right bundle branch block (RBBB). While none of these 8 patients converted to sinus rhythm, there was no difference in baseline QRS width between SR (108 ms (94–119)) and NSR group (109 ms (95–120)), *p* = 0.792. Post-procedural QRS width was similar to baseline QRS (*p* = 0.109 for SR; *p* = 0.08 for NSR).

**Fig. 2.**
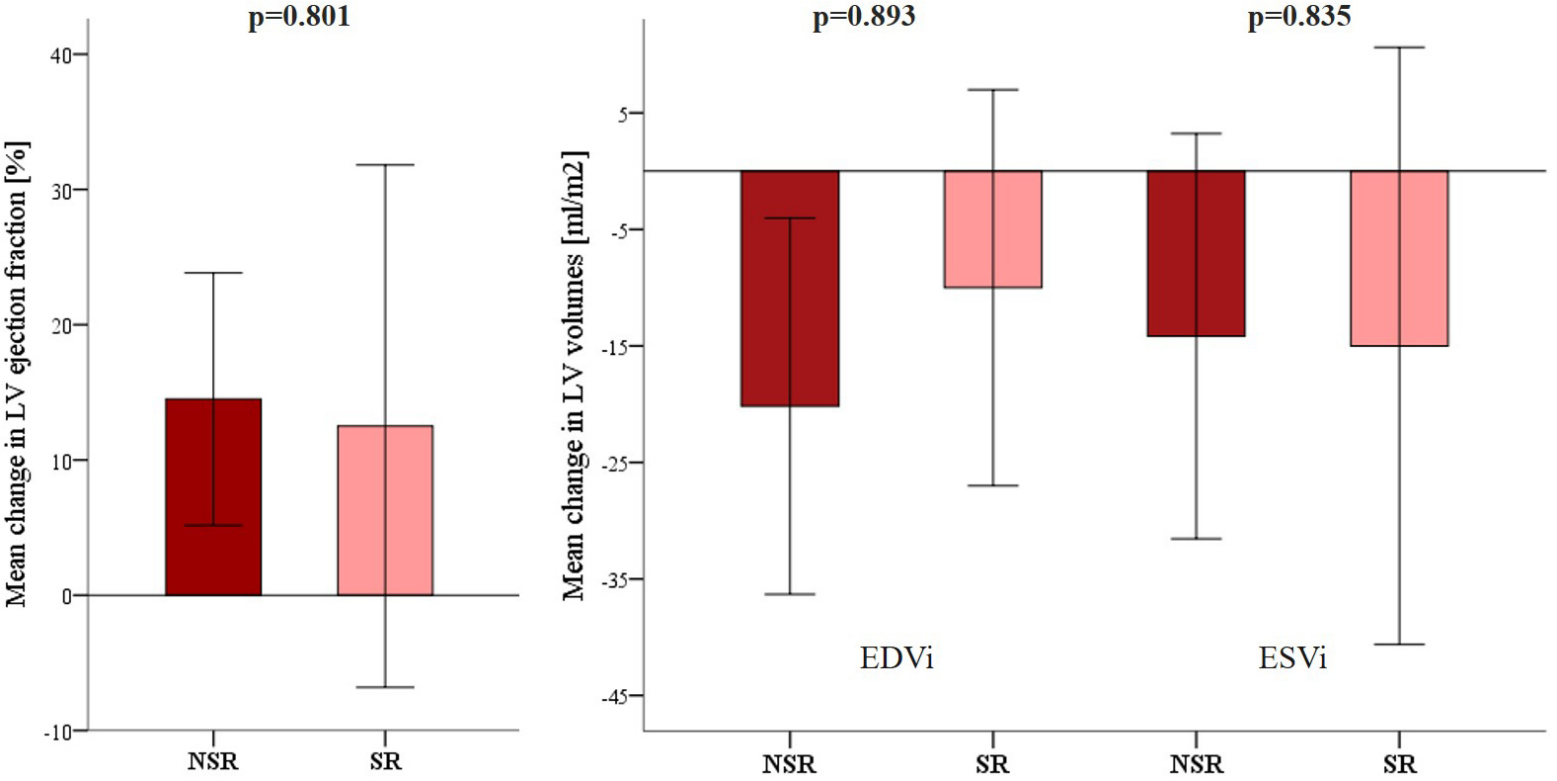
**Comparison of the mean (±SD) changes in echocardiographic left ventricular ejection fraction and volumes between baseline and follow-up according to conversion to sinus rhythm**. Legend: LV, left ventricular; SR, sinus rhythm; NSR, non-sinus rhythm; EDVi, end-diastolic volume index; ESVi, end-systolic volume index.

**Fig. 3.**
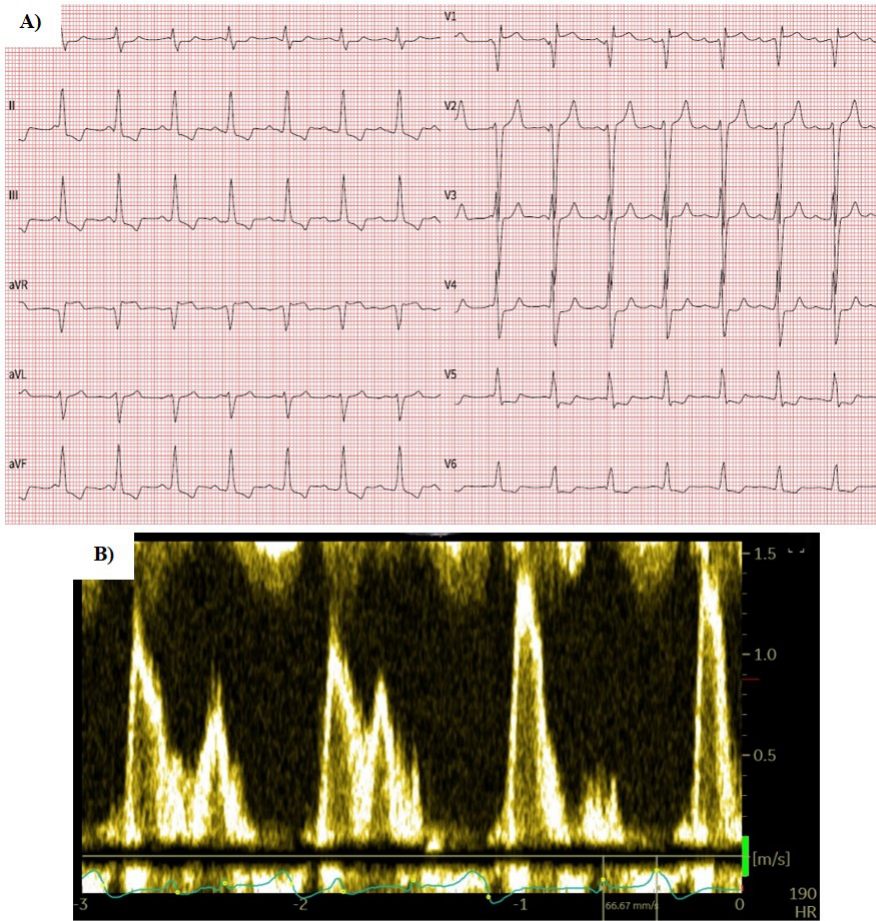
**12-lead ECG (A) and mitral inflow pattern (B) of the patient who converted to sinus rhythm after left bundle branch pacing and atrioventricular node ablation**. Note the VVI pacing mode and atrioventricular dissociation due to the lack of atrial lead. ECG, electrocardiogram.

While there were no significant differences in baseline LVEF (*p* = 1), LVEDVi (*p* = 0.214), and indexed LV systolic volume (LVESVi) (*p =* 0.311), baseline LAVI was, as previously described, significantly smaller in patients who converted to SR (*p* = 0.002) (Table 4). At follow-up, LAVI did not change in any group. The increase in LVEF was numerically comparable in both groups, although, in the SR group, it did not reach statistical significance due to the smaller sample size. Similarly, LVESVi decreased in both groups; however, in the SR group, the decrease did not reach statistical significance due to both smaller sample size and smaller initial volumes. As for LVESVi, we observed consistent changes in LVEDVi. A comparison of mean changes in echocardiographic parameters is presented in Fig. 2. The follow-up electrocardiogram and echocardiographic mitral inflow pattern of the patient who converted to SR after LBBP with subsequent AVNA are presented in Fig. 3.

**Table 4.** **Echocardiographic outcomes of patients by conversion to sinus rhythm at baseline and follow-up**.

	SR (n = 6)	NSR (n = 62)*	*p*-value — comparing groups
Baseline LVEF [%]	40 (±15)	40 (±16)	1
Follow-up LVEF [%]	52 (±8)	50 (±14)	0.671
*p*-value — baseline vs. follow-up	0.174	< **0.001**	
Baseline LVEDVi [mL/$m^{2}$]	56 (51–69)	76 (53–94)	0.214
Follow-up LVEDVi [mL/$m^{2}$]	53 (49–57)	63 (47–82)	0.228
*p*-value — baseline vs. follow-up	0.144	**0.001**	
Baseline LVESVi [mL/$m^{2}$]	32 (27–41)	47 (27–63)	0.311
Follow-up LVESVi [mL/$m^{2}$]	25 (25–26)	29 (21–46)	0.184
*p*-value — baseline vs. follow-up	0.176	< **0.001**	
Baseline LAVI [mL/$m^{2}$]	45 (41–51)	60 (52–75)	**0.002**
Follow-up LAVI [mL/$m^{2}$]	48 (44–54)	66 (52–77)	**0.018**
*p*-value — baseline vs. follow-up	0.345	0.508	

Legend: SR, sinus rhythm group; NSR, non-sinus rhythm group; LVEF, left ventricle ejection fraction; LVEDVi, left ventricular end-diastolic volume index; LVESVi, left ventricular end-systolic volume index; LAVI, left atrial volume index. * In the NSR group follow-up, LVEF value was available in 56 patients and left ventricle volumes were available in 59 patients at baseline and 56 patients at follow-up. Bold values denote statistical significance at the *p* < 0.05 level.

## 4. Discussion

To the best of our knowledge, this is the first study reporting the incidence of spontaneous conversion to SR after CSP and AVNA in patients with refractory symptomatic AF. The main finding of the present study was that spontaneous conversion to SR after AVNA combined with CSP is not uncommon, as it occurred in 8.8% of the patients. Smaller LAVI was identified as the only independent predicting factor for SR restoration in patients undergoing this treatment option.

According to guidelines, antiarrhythmic drugs or PVI are considered to restore and maintain SR as part of rhythm control management [24]. Success rates of rhythm control strategy in patients with paroxysmal AF seem to be better than in patients with persistent AF, where approximately 50% SR maintenance is achievable, according to the literature [25, 26]. As the ‘pace and ablate’ strategy is considered only as a rate control strategy, SR restoration is not anticipated [4]. Nonetheless, AVNA and BiV pacing has been associated with spontaneous reversions to SR in patients with persistent AF, ranging from 7% in one report [7] and 10.3% in the other [9]. In our study, spontaneous SR restoration during follow-up was registered in 6 patients (8.8%), predominantly in patients with smaller initial LAVI. This is in line with the previous BiV study, where LA diameter <50 mm, pacing QRS width, and AVNA were predictors of SR restoration in patients with permanent AF after BiV [9]. Some electrical and morphological changes in atrial structure appear to be reversible upon improvement of cardiac function, decrease in sympathetic activation, and reduction of atrial pressure associated with rate control and regularization after AVNA [1, 2]. In addition, CSP, as the most physiological pacing modality, might further contribute to the preservation or improvement of LV function in these patients. Therefore, it is reasonable to assume that patients with smaller atria have a potential for SR restoration after the ‘pace and ablate’ strategy when CSP is adopted as a pacing modality.

Several beneficial clinical outcomes of CSP modalities combined with AVNA have already been published [16, 17]. In our study, symptomatic improvement was achieved in both groups: 75.8% of patients in the NSR group and all patients in the SR group improved for at least one functional class. Similarly, the number of patients receiving loop diuretics decreased in both groups, however not significantly in the SR group. This difference could be explained by a smaller sample size of the SR group. Echocardiographic outcomes of the ‘pace and ablate’ strategy in this study resemble those mentioned in the previously published literature [14, 15, 17, 27]. LV volumes and LVEF improved in the NSR group. Similar, although not statistically significant, improvement of LVEF and reduction of LV volumes was observed in the SR group. The mean change of LVEF and LV volumes between both groups did not differ. There are several reasons that could be attributed to these findings. First, as atrial leads were not implanted in patients with SR, the patients did not gain any additional benefit from restored atrioventricular (AV) synchrony (Fig. 3). Furthermore, the SR group was numerically smaller with smaller, albeit not statistically, initial LV volumes which might have influenced the power of statistical analysis.

### 4.1 Clinical Implications

Larger studies are warranted to clarify the predicting variables of SR restoration in patients scheduled for an “ablate and pace” strategy with CSP. The incidence of SR restoration is clinically important as these patients may require an upgrade to a dual-chamber device to ensure AV synchrony that could further maintain SR and prevent potential pacemaker syndrome. On the contrary, initial atrial lead implantation in all patients may imply an unnecessary increase in lead burden. Therefore, the ability to identify which patients are expected to experience SR during follow-up is certainly important to optimally manage these patients.

### 4.2 Study Limitations 

The retrospective design of this single-center study with a relatively small number of included patients limits the strength of our findings. Furthermore, a smaller sample size in the SR group might have affected the outcomes compared to NSR. The potential exclusion of patients with severe valvular disease might have had an impact on the results of the study, while the probability of SR restoration in these patients is very low. However, none of the patients were, in fact, excluded due to this exclusion criterion. The study only recorded SR restoration in the device clinic during regular follow-ups that were documented on 12-lead ECG, while potential intermittent conversions to sinus rhythm were not detected. However, persistent sinus rhythm was not achieved in the NSR group. The data on the duration of AF before the ‘pace and ablate’ intervention could not be obtained in several patients. As some of the patients who converted to SR were urgently admitted to the hospital due to acute decompensated HF, a possible shorter duration of AF in these patients could have resulted in less structural and electrical left atrial changes and increased the likelihood of the SR restoration [1, 2, 3]. Therefore, our findings should be interpreted with caution and need to be confirmed in larger studies with longer follow-ups.

## 5. Conclusions

Spontaneous conversion to SR after AVNA combined with CSP is not uncommon, especially in AF patients with smaller left atria. Further studies are warranted to clarify which patients should be considered for an initial dual-chamber device to provide AV synchrony in case of SR restoration.

## Data Availability

The data presented in this study are available upon request from the corresponding author and are not publicly available due to ethical issues.
